# Diseases of the Intestines

**Published:** 1906-04-21

**Authors:** 


### PROGRESS IN MEDICINE AND SURGERY.

### DISEASES OF THE INTESTINES.

**Chronic Constipation.**—A very interesting and instructive debate on the causes and treatment of this condition has been reported.[1] Various points of view, both in the causation and treatment, were advanced, often widely different, but not necessarily contradictory, owing to the manifold nature of the complaint, and to the necessity which each speaker felt in confining his remarks to the range of his own particular experience. The debate was opened by Arbuthnot Lane, who advanced his views with emphasis and originality. His principal points were that in the first place very definite anatomical changes are induced in the abdominal viscera by chronic constipation, which, though obvious to the operating surgeon, who sees the parts during life, may easily be overlooked by the pathologist whose experience is limited to the altered conditions of the deadhouse. He next dilated on the many consequences, often very serious, which follow from these anatomical derangements; and lastly, he briefly outlined his operative treatment by means of anastomosis between the lower end of the ileum and the sigmoid or rectum. The details of the changes produced by chronic constipation are, Lane asserts, somewhat as follows:—Owing to the distension of the large gut by accumulated fæces and flatus the transverse colon descends, and is only held up at the splenic and hepatic flexures by its stronger attachments. This leads to kinking at these angles, the distended cæcum falls into the true pelvis, the sigmoid becomes straightened and lengthened, the ileocæcal aperture is also kinked and obstructed. and the small intestine in turn becomes distended. These changes are followed or accompanied by a chronic peritonitis, with some effusion (comparable to that seen in malignant disease), which leads to the formation of bands and adhesions fixing the parts in their faulty positions. The condition, Lane thinks, is mainly attributable to errors in diet, often occurring in infancy and childhood, as witnessed by the "pot belly," which is often seen accompanying chronic constipation. The results of the faulty anatomical condition set up are:—Pressure of the cæcum on the pelvic organs leading (in women) to uterine and ovarian trouble and to sterility; hydrocele and hernia from the increased intra-abdominal pressure; dilatation of the stomach, adhesions about the appendix, secondary appendicitis, and dragging on the kidneys, especially the right, rendering them movable. The absorption of toxins from the loaded gut produces loss of vigour and premature senility, pigmentation and atrophic conditions of the skin, much absorption of fat, a predisposition to infections of all kinds, and much mental depression. He claims excellent results from his operation, and states that no inconvenience ensues on the exclusion of the colon from the alimentary tract. Although Lane's views are not universally accepted, many subsequent speakers gave evidence as to the correctness of his observations on the anatomical conditions. Goddard, in supporting his main contentions, quoted certain very important experiments he had made to determine the functional importance of the colon. His conclusions were that after excluding the colon, a trifle more (12 per cent.) of the fat consumed in food was discharged in the fæces, that the function of absorbing water and rendering the motions semisolid was soon taken on by the small intestine, and that the only loss which was entailed was that of a small amount of cellulose, which the colon normally digested and absorbed. F. J. Smith, while admitting that Lane's account of the chronic peritonitis, adhesions and dropping of the transverse colon was substantially correct, did not think them due to chronic constipation. He had often seen thoracic adhesions in these cases, and thought their causation at present obscure. He was also of opinion that the appendix was not much influenced by chronic constipation. This difference of opinion is important, as Lane maintains that when appendicular trouble is really caused by chronic constipation with adhesions and inflammation in the cæcum, appendectomy fails to give other than the temporary relief due to rest in bed and careful regulation of the bowels. F. J. Smith's most pertinent criticism, however, was in the stress which he laid on the comparative newness of Lane's operation. He very rightly insisted that before any adequate opinion could be formed, observations must be made on the whole after-life of numerous patients to see whether any serious inconvenience attended the functional elimination of the colon, and whether the relief of the constipation was permanent. The sole interest of the discussion, however, was not confined to Lane's operation. Dickinson contributed a useful classification of the causes of chronic constipation, which he divided into intrinsic and extrinsic. The former, the larger and more important group includes muscular and glandular atony, hepatic insufficiency, chronic colitis, and mechanical causes. The latter include two factors—hard water and improper diet. The views of different authorities as to treatment are interesting in the extreme. Dickinson, for muscular atony, prescribes skilled massage and electricity, combined with such drugs as cascara, aloes, and strychnine; while for the senile cases, where secreting power is deficient, he advises magnesium sulphate, and carbonate with gentian. He also believes in the efficacy of peptenzyme. Palmer, on the other hand, thinks that drugs and massage are both comparatively valueless; and Collier also holds that much "humbug" has been talked about the latter. He believes that very ordinary rubbing is quite good enough, and that there is no necessity for special skill or care in manipulating the abdomen. Goddard, however, maintains that any kind of massage is liable to increase the peritoneal adhesions which occur in chronic constipation, and even when "skilled" is likely to do more harm than good. This is very likely true, but the fallacy probably lies in supposing that in all cases of constipation which can be classified as chronic the same anatomical changes occur. The bulk of evidence in favour of some sort of massage to strengthen at any rate the abdominal parieties is too great to give way before what is, after all, a partly theoretical objection. Equally diverse are the opinions expressed as to the value of a bulky, irritating, indigestible diet in chronic constipation. Palmer thinks that porridge and wholemeal bread are likely to irritate an already inflamed bowel, and set up ulceration above the fæcal accumulations; Dickinson does not appear to support the view that an increased indigestible residue in the food is desirable, while Leonard Williams holds that for every case in which such a line of treatment succeeds, it fails in at least a dozen. Collier, on the other hand, thinks an irritating diet of great service, and recommends as much as five or six apples a day in the summer, and plenty of well-masticated nuts. There seems very little reason for withholding irritating or indigestible matter in certain cases, and though Leonard Williams prefers a drug which will act in a similar manner, it must be remembered that patients will often prefer, and consequently adhere, to a line of treatment which does not involve the taking of pills and medicines, and that in certain cases drugs undoubtedly cease to be efficacious after a time, whereas a dietetic treatment often is continuously successful for years. Turning from these discrepancies of opinion, there is remarkable agreement as to the necessity for a liberal allowance of fluid in the diet of constipated persons. Collier points out that many persons who take habitually a glass or two of wine with their meals seldom take any other liquid, and hence fail to supply the bowel with sufficient water. As to the constipating effects of a hard water, too, all authorities seem agreed. Many speakers praised the large enemata of water or olive oil, Collier also recommending that the latter should be taken by the mouth and used in cooking. He has found this a useful measure in dealing with the poorer patients, who cannot afford expensive drugs or elaborate treatment. Leonard Williams, however, thinks that injections of olive oil should not be given alone. Their action in softening and displacing large fæcal accumulations may lead to the liberation of large amounts of toxin. To neutralise the toxin he always adds 5 or 10 drops of eucalyptus oil to the enema, and gives small doses of calomel by the mouth. Although varied opinions are held as to the newer synthetic purgatives, such as phenol thallein and the like, there is very little doubt about the value of old and well-tried drugs. Leonard Williams has described his method of imitating the "Spa" treatment at home.

He prescribes for the younger and more robust patients a mixture containing mag. sulph. grs. 30, mag. carb. grs. 10, tinc. nucis. vom. ♏ 4, ess. meth. pip. ♏ 10, inf. gent. co. ad. ℥ 1. This he gives three times a day, half an hour before food, followed by a tumbler of hot water. For patients who would find this too depressant he gives mag. sulph. grs. 30-40, acid. sulph. dil. ♏ 1, liq. strych. ♏ 5, ess. menth. pip. ♏ 20, inf. gent. co. ad. ℥ 1, three times a day after food, followed by a tumblerful of water, and adds quinine, iron, or arsenic if required by the condition of the patient. By this means he maintains that frequent small doses of salines are secured, that sufficient liquid is taken, and that the muscular wall is stimulated to carry off the waste products which are dissolved in the liquid contents of the intestine. The addition of the nux vomica he considers of especial importance, as without some such aid to peristalsis the dissolved toxins are merely reabsorbed. By degrees he reduces the drugs till the bowels act regularly on three tumblerfuls of water a day. During the first week of treatment he gives also small doses of calomel ($\frac{1}{3}$-$\frac{1}{6}$ grain) once a day, and afterwards once or twice a week. For young and fairly vigorous patients he finds this line of treatment nearly always succeeds. Palmer, though not on the whole advising the use of purgatives in chronic conditions, strongly recommends strychnine in cases of muscular atony. He believes, however, that it is most certainly an aphrodisiac and hence may be inadvisable in certain cases. In the moderate doses in which Leonard Williams prescribes it, however, any secondary effect of this kind must be very unusual. An interesting contribution to the discussion was that of Campbell Thomson on the influence of the nervous system and its disorders on chronic constipation. He began by pointing out the importance of the muscular walls of the intestine as an organ of expulsion, and the close association which Pawlow has shown to exist between the nervous system and the secretory powers of the glands, as illustrated in the "psychic" secretion of gastric juice. That the cerebral cortex is associated with the secretory activity of the intestines is shown by the abnormal hunger and thirst noted in some cases of cerebral tumour, especially of the temporo-sphenoidal and parietal lobes. He even thinks it possible that "psychic" influence may bear on the pancreatic secretion, and though admitting that higher centres have but small influence on the succus entericus, yet notes the "paralytic" secretion which takes place when a loop of intestine is cut off from all higher control by section of its nerves. He then pointed out the great diminution of secretion which takes take when the musculature of the bowel is paralysed by peritonitis or obstruction, and said that he had little doubt that less marked diminution also occurred when the bowel was partially paralysed or in a condition of atony. Then the ileocæcal valve had, he said, been recently shown to be not merely a mechanical valve, but a definite sphincter, controlled by nerves and comparable to the pylorus. That the higher centres should not interfere with the normal action of the lower centres in regulating the evacuations was an important but often unattainable desideratum; but he held that suitable drugs could generally counteract the bad effects of worry, excitement, and fatigue on the cerebral cortex. In conclusion he dwelt on the reverse picture, the bad effects on the cerebral cortex which occurred when, owing to chronic constipation, much toxin was absorbed and local changes were produced by the pressure and distension of the intestinal walls on the nerve endings, which thus were constantly sending disturbing influences to distant parts of the nervous system.

[1]Clin. Jour., Aug. 23 and 30, 1905.

